# Treatment of mucocutaneous manifestations in Behçet’s disease with anakinra: a pilot open-label study

**DOI:** 10.1186/s13075-017-1222-3

**Published:** 2017-03-24

**Authors:** Peter C. Grayson, Yusuf Yazici, Melissa Merideth, H. Nida Sen, Michael Davis, Elaine Novakovich, Elizabeth Joyal, Raphaela Goldbach-Mansky, Cailin H. Sibley

**Affiliations:** 1 National Institutes of Health, NIAMS, Bethesda, MD USA; 2 New York University, New York, NY USA; 3 Oregon Health & Science University, 3181 SW Sam Jackson Park Rd OP-09, Portland, OR 97239 USA

**Keywords:** Vasculitis, Behçet’s disease, Anakinra, Autoinflammatory disease, Clinical trial

## Abstract

**Background:**

The effect of IL-1 blocking therapy on mucocutaneous manifestations of Behçet’s disease is incompletely understood.

**Methods:**

Six patients with Behçet’s disease and ongoing oral/genital ulcers for ≥1 month were enrolled into an adaptive, two-phase clinical trial and included in the analysis. Study duration was 6 months with extension up to 16 months. All were treated non-blinded with anakinra 100 mg subcutaneous daily with the option to escalate the dose to 200 mg in partial responders after 1 month and 300 mg after 6 months. Patients recorded the number and severity of ulcers in daily diaries. The primary outcome was remission defined as no ulcers on physical exam for two consecutive monthly visits between months 3 and 6. Secondary outcomes included the number and severity of patient-reported ulcers, patient/physician global scores, and standardized disease activity scores.

**Results:**

Two of six patients achieved the primary outcome. Five of six patients had improvement in the number and severity of ulcers. Non-statistically significant improvements were seen in secondary outcomes. Over the entire study, patients reported ≥1 oral and ≥1 genital ulcer on 665 (66%) and 139 (14%) days, respectively. On anakinra 200 mg vs 100 mg, patients reported fewer days with oral ulcers (65% vs 74% of days, *p* = 0.01) and genital ulcers (10% vs 22% of days, *p* < 0.001) and milder oral ulcer severity (*p* < 0.001). Increase of anakinra to 300 mg did not result in further improvements. Adverse events were notable for mild infections.

**Conclusion:**

Anakinra at an optimal dose of 200 mg daily had an acceptable safety profile and was partially effective in the treatment of resistant oral and genital ulcers in Behçet’s disease.

**Trial registration:**

Clinicaltrials.gov. NCT01441076. Registered on 24 September 2011.

## Background

Behçet’s disease (BD) is a multisystem inflammatory disease characterized by oral ulcers, genital ulcers, pustular or nodular skin lesions, uveitis or retinal vasculitis, and pathergy. The cardinal manifestation of BD is oral ulceration. Despite well-proven, effective therapies for mucocutaneous manifestations [1], many patients continue to exhibit refractory oral and genital ulcerations.

The relative importance of innate and adaptive immunity in the pathogenesis of disease is incompletely understood with most evidence suggesting joint roles. IL-1 is a central cytokine in innate immunity and its blockade is often effective in the treatment of autoinflammatory diseases caused by disruptions of innate pathways. Case reports and series suggest efficacy of IL-1 blockade in mucocutaneous disease [2–5]. Furthermore, initial promising results with gevokizumab - a monoclonal IL-1β blocking antibody - in inflammatory eye disease has generated further interest in IL-1 blockade in this disorder, although notably, mucocutaneous flares did occur in this study [6].

While there are encouraging data on the use of IL-1 blockade in the treatment of mucocutaneous BD, so far these have all been from retrospective studies or case reports. The objectives of this two-phase, adaptive study design were to evaluate the safety and efficacy of IL-1 blockade with the IL-1 receptor antagonist anakinra, in controlling oral and genital ulcers in an American cohort of patients with refractory mucocutaneous manifestations of BD.

## Methods

### Patients

Adult patients with BD as defined by the International Study Group Criteria were eligible for participation. The trial was a single-center study conducted at the National Institutes of Health (Bethesda, MD, USA). Patients were recruited by study staff from an observational cohort of patients with BD from February 2012 to February 2014. Patients had active mucocutaneous disease defined by physician documentation of at least one oral or genital ulcer in the month prior to enrollment with direct observation of an ulcer at one month prior to enrollment and also at the time of enrollment. Patients were on a stable or decreasing dose of steroids, nonsteroidal anti-inflammatory medications, colchicine, or disease-modifying antirheumatic drugs for 4 weeks. Exclusion criteria included (1) organ or life-threatening disease including ocular inflammation within 3 months; (2) treatment with tumor necrosis factor inhibitors within 8 weeks; (3) infection including tuberculosis, hepatitis B or C, and human immunodeficiency virus; (4) history of a severe or chronic medical condition including congestive heart failure, malignancy, or uncontrolled asthma; (5) significant cytopenia; and (6) pregnancy or breastfeeding.

### Study design

This study was a two-stage adaptive design. Seven patients could be enrolled in the initial phase. If 5 of 7 patients met the primary outcome of complete response between months 3 and 6, the second study phase would be initiated with a total of 20 patients enrolled into the study. In the second phase, patients meeting complete response criteria would be randomized to continuation of study drug vs placebo for 6 months. If five of seven patients did not meet the complete response criteria by 6 months, patients meeting complete or partial response criteria could be continued on open-label therapy for the remainder of the trial. The full study protocol is listed on Clinicaltrials.gov (NCT01441076).

### Treatments

All patients were initially treated with anakinra 100 mg daily via subcutaneous injections. In other types of autoinflammatory disease, increasing the dose of anakinra above 100 mg per day is often required to obtain disease control [7]. Consequently, if oral or genital ulcers persisted after the first month of treatment, anakinra was increased to 200 mg daily. For patients with ulcers at month 6, anakinra was further increased to 300 mg daily. At each study visit, glucocorticoids could be decreased up to 20% of the total dose per judgment of the investigator.

### Assessments

Study visits occurred at baseline, day 10, month 1, and then monthly up to 16 months. A safety evaluation was performed on day 7 and at all study visits. Patients were evaluated by a multi-disciplinary team including rheumatology, dermatology, gynecology, ophthalmology, oral medicine, and gastroenterology as appropriate. The following questionnaires and disease assessment indices were collected at each visit: physician and patient visual analog scales (VAS), BD current activity form (BDCAF), BD quality of life (BDQOL), and Behçet’s syndrome activity scale (BSAS) [8–10]. Patients maintained daily diaries recording the number and severity of oral and genital ulcers. Severity of ulcers was scored on a scale from absent (0) to severe (4).

### End points

The pre-specified primary outcome was complete response defined as absence of oral and genital ulcers on physical examination on two consecutive monthly visits between months 3 and 6. To meet the complete response criteria, patients could not at any point during the study have evidence of organ-threatening disease or ocular inflammation as defined by the Standardization of Uveitis Nomenclature (SUN) criteria. Partial response was defined as a decrease in the number of genital or oral ulcers at the current and previous study visits compared to the baseline number of ulcers. Treatment failure was defined as lack of complete or partial response at 6 months. Patients not completing the initial phase of the study were considered treatment failures.

The pre-specified secondary outcomes included the number of physician-observed ulcers, number and severity of patient-reported ulcers, patient and physician global scores, and standardized disease activity scores.

### Adverse events

Adverse events were graded according to the National Cancer Institute Common Terminology Criteria for Adverse Events (CTCAE) v4.0. An independent study safety officer met with the principal study investigators every 6 months to review adverse events.

### Statistical analysis

Criteria for proceeding beyond the initial study phase were based on the Simon’s optimal two-stage design. The probability of a response rate worthy of further investigation was set as 80% and the probability of a response rate not worthy of further investigation was 50%. Based on a two-sided significance level of 0.05 and power of 80%, up to 7 patients were needed in the first stage and an additional 13 patients in the second stage; if 5 of 7 patients did not respond during the initial stage, the second stage would be terminated. If 14 of 20 patients responded in the total population, the treatment would be considered successful.

Summary statistics were calculated including mean and median values, ranges, standard deviations, and frequency distributions. The primary outcome was evaluated using descriptive statistics. For the secondary outcomes, two-sample comparisons were performed with the Wilcoxon signed rank test for paired continuous measures and Fisher’s exact test for binary measures. Patients’ diary data were analyzed using repeated-measures analysis of variance to account for within-subject correlation. Severity of ulcer pain was assessed by the comparing the percentage of days in which the patient-reported severity score was 1 vs >1.

## Results

### Patient characteristics

Six patients were recruited into the initial study phase. Baseline clinical and demographic characteristics are listed in Table 1. All patients had a longstanding history of both oral and genital ulcers. The majority were female ((n =5) and Caucasian (n = 4) and only one was human leucocyte antigen (HLA)-B51 positive. Two patients were taking glucocorticoids. The number of patients with organ involvement was as follows: cutaneous (n = 6), musculoskeletal (n = 6), gastrointestinal (n = 3), ocular (n = 3), vascular (n = 3), and neurologic (n = 0). At baseline, all patients had at least one oral ulcer (range 2–5) and two had genital ulcers (2 and 3 ulcers, respectively).

**Table 1:** Patient characteristics at enrollment

Patient	Age (years)	Sex	Country of origin	Race	Relevant prior treatment	Current treatment	Historical disease features	Age at ulcer onset (years)	Primary outcome
1	59	F	USA	Caucasian	Abatacept Adalimumab Azathioprine Colchicine Glucocorticoids Infliximab	Azathioprine	**Oral and genital ulcers**, GI ulcers, **skin pustules**, **arthralgias**	7	Treatment failure
2	19	F	USA	Caucasian	Colchicine Glucocorticoids Sulfasalazine	Colchicine Sulfasalazine Prednisone (15 mg/day)	**Oral and genital ulcers**, GI ulcers, thrombophlebitis, **arthralgias, skin pustules**, folliculitis, pathergy, **HLA-B51-positive**	16	Complete response
3	40	F	USA	Mixed	Colchicine, Hydroxychloroquine Glucocorticoids	Colchicine Hydroxychloroquine	**Oral and genital ulcers**, **skin pustules, arthritis**	38	Partial response
4	30	F	USA	Caucasian	Colchicine Glucocorticoids	None	**Oral and genital ulcers, skin pustules**, anterior uveitis, arthritis	2	Complete response
5	26	F	USA	Caucasian	Azathioprine Glucocorticoids	Azathioprine	**Oral and genital ulcers**, GI ulcers, **skin pustules**, deep vein thrombosis	21	Treatment failure
6	36	M	Ethiopia	African	Colchicine Glucocorticoids	Prednisone (20 mg/day)	Oral and genital ulcers, skin pustules, panuveitis, arthritis	31	Partial response

Active disease features at the time of enrollment are highlighted in bold

### Efficacy assessments

Two of six patients met the primary outcome of complete remission by month 6 (Table 1). Two patients met the criteria for partial response. Two patients were considered treatment failures. One patient terminated the study at month 5 due to ongoing arthralgia and pustular skin disease. The other patient terminated the study at month 3 due to persistent oral ulcers and arthritis. Given the low rate of complete remission, a seventh patient was not recruited into the initial phase, and the randomized withdrawal phase was not conducted. Patients who had complete or partial response (n = 4) continued on open-label therapy for the remainder of the trial (range 12–16 months).

The daily dose of anakinra was increased from 100 mg to 200 mg in all patients at month 1. Anakinra was further increased to 300 mg daily in three patients who participated in the extended follow-up phase of the trial. Daily prednisone requirements were reduced in both patients who were on glucocorticoids at enrollment. Out of six patients with musculoskeletal symptoms, improvement in joint pain was noted in four patients; however, no patient experienced complete resolution of symptoms throughout the trial. Out of four patients with active skin lesions at the baseline visit, improvement was noted in two patients. No patient experienced organ or life-threatening disease activity, including ocular inflammation, during the trial.

**Fig. 1:**
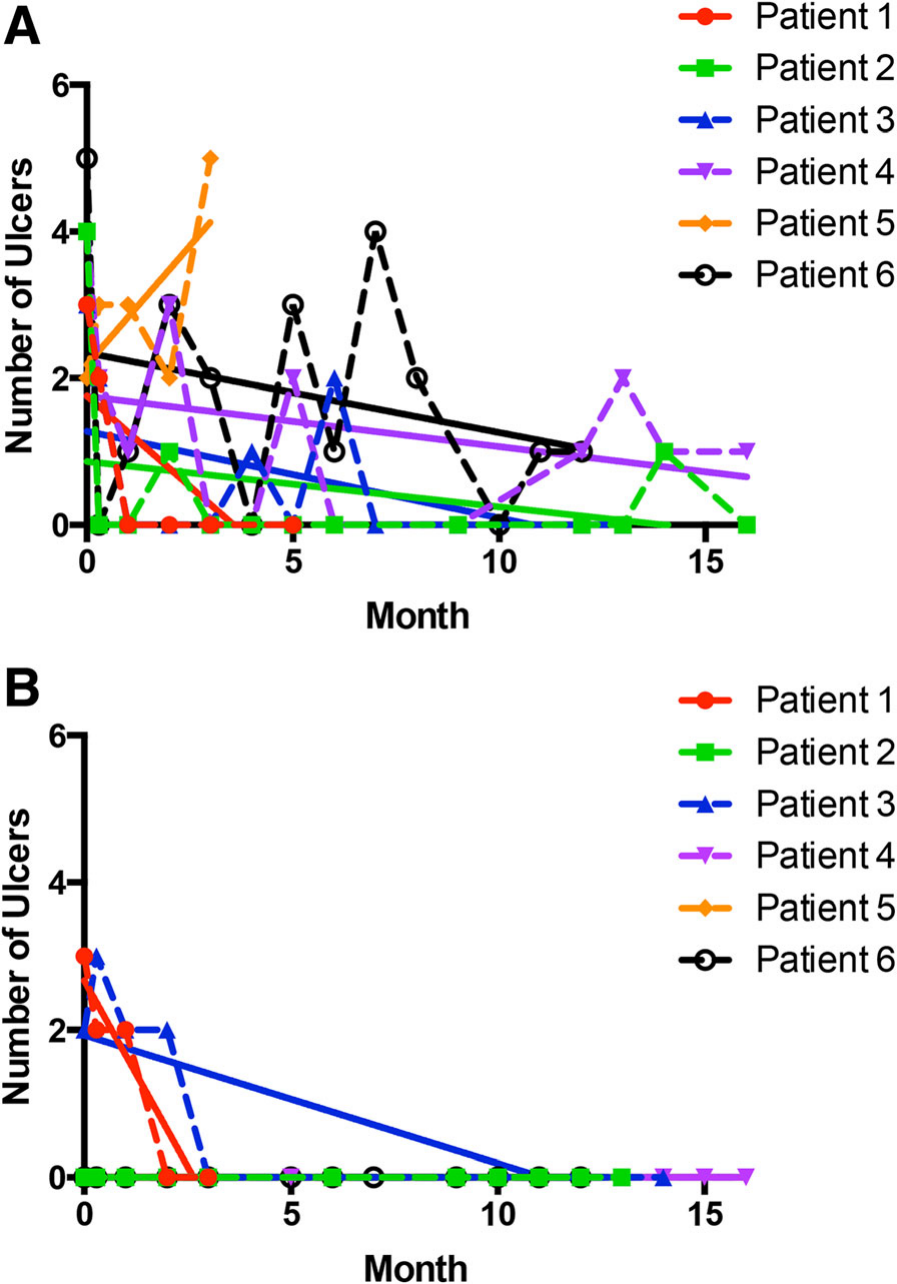
Physician-observed oral and genital ulcers. The number of physician-observed oral ulcers (**a**) and genital ulcers (**b**) are shown for each patient at each study visit with trend lines (*solid lines*). Five of six patients had improvement in the frequency of ulcers over the course of the trial

The number of physician-observed oral and genital ulcers at each study visit is depicted in Fig. 1. Oral ulcers improved in five of six patients. All patients who were either complete or partial responders had at least one new physician-observed oral ulcer after month 6. Genital ulcers resolved by month 3 in the two patients with genital ulcers at baseline and no further genital ulcers were observed by a physician in any patient.

Secondary outcomes are shown in Table 2. Non-significant improvements occurred in all secondary outcome measures, including the number of physician-observed oral and genital ulcers, patient global VAS, BDCAF, BSAS, and BDQOL scores.

**Table 2:** Secondary outcome measures

	Baseline (n = 6)	Month 1 (n = 6)	Month 2 (n = 6)	Month 3 (n = 6)	Month 6 (n = 4)	Month 9 (n = 4)	Month 12 (n = 4)
Oral ulcer number	3.5 (2–5)	0.5 (0–3)	1.5 (0–3)	1.5 (0–5)	0.5 (0–2)	0 (0–1)	0.5 (0–1)
Genital ulcer number	0 (0–3)	0 (0–2)	0 (0–2)	0 (0)	0 (0)	0 (0)	0 (0)
Physician global VAS (0–100)	16.5 (12–79)	13 (6–39)	18.5 (10–32)	14.5 (4–23)	8.5 (7–24)	7.5 (1–58)	14 (3–30)
Patient global VAS (0–100)	73.5 (9–90)	32 (3–92)	48 (0–95)	68.5 (14–88)	51.5 (3–91)	22 (14–53)	35 (23–55)
BDCAF (0–12)	6 (5–11)	6.5 (3–8)	5 (3–8)	5.5 (3–10)	5 (4–6)	5 (1–6)	6 (3–7)
BSAS (0–100)	46 (22–80.5)	34 (19.5–49)	42 (20.5–58)	24.7 (9.5–52)	21.3 (0–44)	24.5 (8.5–30)	33 (15–57)
BDQOL (0–30)	16 (10–24)	10.5 (0–21)	10 (1–23)	14 (0–22)	9.5 (0–22)	6 (3–14)	14 (6–15)

All results are presented as median (range). *VAS* visual analog scale, *BDCAF* Behçet’s Disease Current Activity Form, *BSAS* Behçet’s Syndrome Activity Score, *BDQOL* Behçet’s Disease Quality of Life

**Fig. 2:**
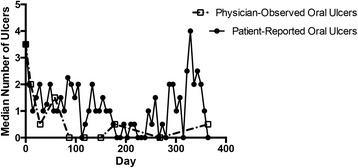
Patient-reported versus physician-observed oral ulcers. The median numbers of physician-observed oral ulcers at each study visit (*dotted line*) are presented in comparison to the number of patient-reported oral ulcers (*solid line*) throughout the trial. Patient-reported oral ulcers are presented as the median number of oral ulcers recorded in patients’ daily diaries throughout each week of the trial

In patients’ daily diaries all patients noted at least one new oral ulcer during the course of the trial and three patients noted at least one new genital ulcer. Presence of oral ulcers was reported on 665 of 1005 study days (66%). With anakinra 200 mg vs 100 mg, four of six patients reported fewer days with oral ulcers (65% vs 74% days, respectively, *p* = 0.01) and all reported milder ulcer severity (*p* < 0.01). Presence of genital ulcers was reported on 139 of 1005 study days (14%). With anakinra 200 mg vs 100 mg, two of three patients reported fewer days with genital ulcers (10% vs 22% days, respectively, *p* < 0.01 with a non-significant decrease in severity (*p* = 0.08)). Dose escalation to 300 mg did not result in fewer oral or genital ulcers or milder ulcer severity. Patients’ daily diaries documented a greater burden of oral ulcers throughout the trial than the number of ulcers observed by a physician at each study visit (Fig. 2).

### Adverse events

There were four serious adverse events, which occurred in the same patient. This patient was diagnosed with pulmonary arterial hypertension during the trial; however, onset was suspected prior to enrollment. Injection site reactions occurred in three patients within the first month of the trial and resolved with supportive measures. There were several non-serious infections, predominantly upper respiratory and yeast infections. There was no observed relationship between the frequency of adverse events and the dose of anakinra Table 3.

**Table 3:** Adverse events

	Events	Incidence rate (per 100 days)
Anakinra 100 mg/day (194 total days)		
*Serious events*		
Hospitalized to rule out thrombus	1	0.5
*Non-serious events*		
Injection site reaction	3	1.5
Oral thrush	1	0.5
Other	2	1.0
Anakinra 200 mg/day (1371 total days)		
*Serious events*		
Pulmonary artery hypertension	1	0.1
Pre-syncope	1	0.1
Non-cardiac chest pain	1	0.1
*Non-serious events*		
Upper respiratory infection	5	0.4
Alopecia	2	0.2
Edema	2	0.2
Vaginal yeast infection	1	0.1
Other	9	0.7
Anakinra 300 mg/day (322 total days)		
*Serious events*		
• None	0	0
*Non-serious events*		
• Upper respiratory infection	2	0.6
• Vaginal yeast infection	1	0.3
• Other	2	0.6

## Discussion

BD is a heterogeneous disorder with clusters of disease and ethnic subtypes. Conflicting data are emerging on the efficacy of IL-1 blockade in this disorder. In this study, we systematically treated patients with oral and genital ulcers with increasing doses of anakinra and reported standardized clinical outcomes.

BD shares clinical features with autoinflammatory diseases, including the lack of autoantibodies, periodic episodes of inflammation, mucosal surface involvement, and neutrophilic infiltration. The prototypical cytokine in autoinflammation is IL-1 and blockade of this cytokine is highly effective for many autoinflammatory diseases. High levels of IL-1β have been observed in serum and synovial fluid from patients with BD [11, 12], and polymorphisms in IL-1 are reported in some cohorts of patients, although this was not confirmed in a larger gene-wide association study [13].

Case reports and series suggest a mixed effect of IL-1 blockade on clinical manifestations of BD. The largest series to date is a retrospective multicenter study of 30 patients with varied manifestations of BD treated with either anakinra 100 mg daily or canakinumab 150 mg every 6–8 weeks; 13 of 30 patients achieved complete remission after 12 months [4]. For mucocutaneous disease alone, an additional series described a rapid improvement in nine patients treated with anakinra 100 mg daily; however, relapses were common ultimately resulting in a poor response in seven patients [5]. A smaller series of three patients with refractory mucocutaneous disease reported a prompt and sustained response to canakinumab in all patients [3]. For eye disease, the most promising study was an open-label study of seven patients with refractory disease treated with gevokizumab [6]. Unfortunately, initial optimism was tempered by a phase III multicenter study, EYEGUARD-B, which failed to reach its primary endpoint.

We prospectively demonstrated a partial response of oral and genital ulcers in BD to anakinra. This was evidenced by objective measurements of ulcers and standardized disease activity outcomes. It is possible that our strict complete response criterion of no ulcerations on two separate visits may have been too stringent, as improvement in refractory disease may be clinically relevant even without a complete response. This is underscored by the recent positive results for apremilast in the treatment of mucocutaneous BD, for which the primary study outcome was reduction in the number of ulcers rather than sustained complete response [14]. While our criterion was chosen based on prior pivotal trials [15], the development of valid and widely accepted outcome measures is an active area of research in BD [16]. Anakinra was well-tolerated and adverse events were few.

Patient-reported outcomes and daily diaries were used to capture different elements of disease activity. IL-1 blockade reduced the number and severity of ulcers for both physician-based and patient-reported outcomes. These findings may be more relevant than a complete response, given that a complete response is seldom observed, even in successful studies. The physician-observed ulcer burden was relatively minimal after baseline. In contrast, in the periods between physician-monitored visits, patients recorded a substantial ulcer burden in daily diaries. Future clinical trials in BD should strongly consider incorporating patient-reported outcomes into the trial design, as perceptions of illness may differ between patients and physicians.

We escalated the dose of anakinra beyond the standard Food and Drug Administration (FDA)-approved dose of 100 mg daily for treatment of rheumatoid arthritis. We feel this is critical as high doses are often necessary in other autoinflammatory diseases, with doses up to 10 mg/kg having an adequate safety profile [17]. As we observed improvement from anakinra 100 mg to 200 mg daily but no further improvement with further escalation, we determined that the optimal dose is 200 mg daily and our study did not suffer from inadequate dosing. We studied only patients with active mucocutaneous findings and do not advocate anti-IL-1 treatment for more severe manifestations of disease.

Limitations include that our study was small, unblinded, and ultimately single-arm. However, by using a two-phase adaptive design, we were able to reach our conclusions while minimizing the cost and effort that would be required for a larger study. While there was a difference in degree of clinical response to anakinra among patients in this trial, the small sample size precludes identification of potential subsets of patients who may be more or less responsive to anakinra. Similarly, it also precludes a more thorough evaluation of potential confounders including concomitant medications and important lifestyle factors such as smoking.

Given the relapsing-remitting nature of this disease, an element of benefit could be that we minimized the effects of regression to the mean, by requiring that patients have ongoing active disease with physician-observed ulcers persistent for a month prior to enrollment. This is a similar strategy to that used in a recent phase II study of apremilast in mucocutaneous BD [14].

All patients resided in the USA and the majority was female, which may limit the generalizability of findings to other cohorts. Because of this, we used strict enrollment criteria to improve the homogeneity of our cohort. The release of data on the numbers of oral ulcers in EYEGUARD-B would be helpful to validate these findings in a separate cohort.

Our results confirm those in the literature but with some important differences. Similar to prior studies, most patients benefited from treatment with anakinra, but improvement in disease was neither complete nor sustained. In contrast to prior studies, our study was prospective, it was based on objective measurements of disease activity, and it addressed the issue of insufficient dosing with dose escalation. As some patients did achieve benefit, we concur with case reports that a complete response is possible.

Our results parallel those of apremilast in the treatment of refractory mucocutaneous BD. Apremilast significantly reduces ulcer burden from a mean of 3.0 ulcers at baseline to 0.5 ulcers after 6 months. In the present study, which was not placebo-controlled, we observed a similar magnitude of reduction in oral ulcer burden from 3.5 ulcers at baseline to 1.5 ulcers after 3 months and 0.5 ulcers after 6 months (n = 4 patients) of treatment with anakinra. Differences in trial design may contribute to differences in study populations between these studies. Unlike the apremilast trial, patients taking >10 mg daily prednisone or receiving concomitant immune modulating therapy were not excluded from study participation.

## Conclusion

In summary, anakinra is partially effective for the mucocutaneous manifestations of BD. Anakinra given at doses up to 300 mg daily was well-tolerated with minimal adverse effects. IL-1 blockade can reduce the frequency and severity of ulcerative disease in BD and therefore can potentially be considered in selected patients with treatment refractory mucocutaneous disease. These findings provide additional evidence that innate immune system dysfunction plays a role in the pathogenesis of BD.

## Abbreviations

BD: Behçet’s disease
BDCAF: Behçet’s disease current activity form
BDQOL: Behçet’s disease quality of life
BSAS: Behçet’s syndrome activity scale
CTCAE: common terminology criteria for adverse events
IL-1: interleukin-1
SUN: standardization of uveitis nomenclature
VAS: visual analog scale
